# Editorial for the Special Issue “Pharmacological Activities and Mechanisms of Action of Natural Products”

**DOI:** 10.3390/cimb47090743

**Published:** 2025-09-11

**Authors:** Shining Loo, Antony Kam, Chunyue Du, Simon Ming-Yuen Lee

**Affiliations:** 1 Wisdom Lake Academy of Pharmacy, Xi’an Jiaotong-Liverpool University, Wuzhong No.111, Renai Road, Suzhou 215123, China; 2 Department of Biosciences and Bioinformatics, Xi’an Jiaotong-Liverpool University, Wuzhong No.111, Renai Road, Suzhou 215123, China; chunyue.du@xjtlu.edu.cn; 3 State Key Laboratory of Quality Research in Chinese Medicine, Institute of Chinese Medical Sciences, University of Macau, Macao, China; simon-my.lee@polyu.edu.hk; 4 Department of Food Science and Nutrient, The Hong Kong Polytechnic University, Hung Hom, Kowloon, Hong Kong, China

## 1. Introduction

Natural products have long fascinated scientists as a vast source of structurally diverse bioactive compounds, serving as templates for many contemporary pharmaceuticals. This Special Issue of *Current Issues in Molecular Biology*, titled “Pharmacological Activities and Mechanisms of Action of Natural Products”, features 16 original research articles and reviews exploring the pharmacological profiles of compounds from fungi, medicinal herbs, fruits, higher plants, and purified molecules.

These works employ rigorous mechanistic analyses and therapeutic assessments to show how natural products regulate essential biological processes, such as alleviating oxidative stress, suppressing inflammatory pathways, modulating immune responses, and restoring metabolic balance. This approach not only identifies new therapeutic targets but also accelerates translation from laboratory discoveries to clinical applications. In this editorial, we provide an integrated overview of all 16 contributions, organized thematically to illuminate the current state and future directions in natural product pharmacology.

## 2. Fungal Natural Product Pharmacology

Macrofungi are rich in structurally complex bioactives, especially polysaccharides and triterpenoids [1,2], which display robust antioxidant, immunomodulatory, and antineoplastic effects via mechanisms like reactive oxygen species (ROS) neutralization and apoptosis induction.

Three contributions in this Special Issue:

• Research on wild *Ganoderma lucidum* from high-altitude regions shows that extraction solvent polarity markedly affects bioactive profiles and pharmacological outcomes [3].
• Complementary studies reveal *G. lucidum* extracts’ anti-senescence effects, including reduced oxidative stress and cellular aging, with potential dermatological uses dependent on standardized extraction methods [4].
• A review of *Inonotus obliquus* (chaga mushroom) outlines its anti-inflammatory and antineoplastic mechanisms via Nrf2 and NF-κB pathway modulation. It advocates metabolic engineering for sustainable production, while addressing translational hurdles, and extends to triterpenoids and melanin fractions that boost follicular regeneration under environmental stress (**Contribution 1**).

## 3. Polyherbal Formulation Natural Product Pharmacology

Traditional polyherbal blends often yield synergistic effects surpassing those of isolated components [5,6,7], excelling in anti-inflammatory actions, wound healing, and metabolic regulation through cytokine modulation and enzymatic interventions.

One contribution in this Special Issue:

• A standardized formulation of five Korean medicinal herbs (HRMC5) boosts dermal cell viability, provides UV photoprotection, speeds wound healing, and eases menopausal symptoms, supporting its role in dermatological therapies [8].

## 4. Fruit- and Plant-Derived Natural Product Pharmacology

Fruits and plants offer accessible bioactives [9,10] with proven cardiovascular benefits, achieved through lipid regulation, oxidative stress reduction, and inflammation control, alongside emerging roles in regenerative medicine.

Four contributions in this Special Issue:

• Red watermelon (*Citrullus lanatus*) compounds exhibit atheroprotective effects by inhibiting macrophage lipid buildup and enhancing reverse cholesterol transport, endorsing dietary strategies for cardiovascular prevention [11].
• *Platycladus orientalis* leaf extract promotes follicular regeneration via dermal papilla cell proliferation and canonical pathway activation, suggesting applications in hair loss prevention and growth promotion (**Contribution 2**).
• *Nelumbo nucifera* (lotus) leaf extracts alleviate inflammatory diarrhea in models by suppressing pro-inflammatory mediators and apoptosis through pathway inhibition, positioning them as natural anti-inflammatory agents (**Contribution 3**).
• *Litchi chinensis* (Lychee) pericarp extracts reduce hyperuricemia via xanthine oxidase inhibition and improved renal uric acid excretion, validating their use as functional nutraceuticals (**Contribution 4**).

## 5. Bioactive Molecule Natural Product Pharmacology

Purified natural product molecules enable the precise targeting of disease pathways, offering insights into their roles in inflammation, oxidative stress, metabolism, and cellular protection.

Eight contributions in this Special Issue:

• Compounds like quercetin modulates the phosphomonoesterase activity of protein tyrosine phosphatase nonreceptor type 22, a key immune regulator with implications for cancer and autoimmune therapies (**Contribution 5**).
• Curcumin from *Curcuma longa* attenuates denervation-induced muscle atrophy by regulating inflammatory mediators and calcium homeostasis, building on its established anti-inflammatory profile [12].
• Fraxin, a coumarin glucoside from *Fraxinus* species, curbs inflammatory responses and foam cell formation via targeted pathway inhibition, aiding in vascular pathology management [13].
• Abrin, a ribosome-inactivating protein from *Abrus precatorius*, exhibits paradoxical effects in inhibiting protein synthesis in certain cellular contexts while potentiating immune responses in others, suggesting potential applications in autoimmune disease modulation (**Contribution 6**).
• Notoginsenoside R1 from *Panax notoginseng* confers cardioprotection against ischemia–reperfusion injury through the preservation of mitochondrial integrity and ROS mitigation [14].
• Tauroursodeoxycholic acid (TUDCA), a naturally produced hydrophilic bile acid, protects retinal cells from oxidative damage through autophagy induction, with potential of being an age-related macular degeneration therapeutic (**Contribution 7**).
• 3-Deoxysappanchalcone, a chalcone derived from *Caesalpinia sappan*, mitigates fine particulate matter (PM_2.5_)-induced pulmonary injury through the preservation of endothelial barrier function and inflammatory suppression [15].
• Paclitaxel, a naturally occurring diterpenoid compound extracted from the bark of Pacific yew tree, induces endoplasmic reticulum stress in reproductive tissues, revealing fertility implications that necessitate protective co-therapeutic strategies (**Contribution 8**).

## 6. Concluding Perspectives and Future Directions

This Special Issue vividly illustrates the broad therapeutic promise of natural products from fungi, herbs, fruits, plants, and purified molecules. These agents adeptly target pathways underlying oxidative stress, inflammation, metabolism, and cancer, merging ethnopharmacological heritage with cutting-edge molecular insights. Key takeaways include the need for optimized extraction techniques, standardization, and quality control to ensure consistent efficacy.

Although preclinical data are encouraging, advancing to clinical stages requires robust trials, advanced analytics, and thorough safety assessments. Ultimately, natural products bridge ancient wisdom and modern innovation, offering fertile ground for novel drugs to tackle pressing health challenges [16,17,18].
